# Hydrocephalus owing to ventriculoperitoneal shunt dysfunction

**DOI:** 10.1002/jgf2.525

**Published:** 2022-02-16

**Authors:** Toshimasa Yamaguchi

**Affiliations:** ^1^ 13877 Primary Care and Advanced Triage Section Osaka City General Hospital Osaka Japan

**Keywords:** abdominal radiography, catheter fracture, hydrocephalus, ventriculoperitoneal shunt

## Abstract

A 43‐year‐old woman presented to our hospital with headache accompanied with nausea and intermittent vomiting without abdominal pain. The patient had undergone ventriculoperitoneal shunt placement for hydrocephalus owing to quadrigeminal cistern arachnoid cyst. Cranial computed tomography demonstrated enlarged bilateral ventricles, and the abdominal radiograph demonstrated a reverse U‐shaped catheter that seemed to have been fractured in the left peritoneal cavity.

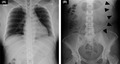

A 43‐year‐old woman presented to our hospital with a 1‐week history of deteriorating headache accompanied with nausea and intermittent vomiting without abdominal pain. The patient had undergone ventriculoperitoneal shunt placement for hydrocephalus owing to quadrigeminal cistern arachnoid cyst at the age of 2 years and shunt revisions at ages 12, 13, and 20 years. The catheter had finally been placed in the right ventricle.

Her Glasgow Coma Scale score was 15; however, she responded slowly to external stimuli. Her blood pressure was 138/78 mmHg, pulse rate was 72 beats/min, and respiratory rate was 20 beats/min. Further physical examination revealed that she had no fever, vision loss, anisocoria, meningeal sign, or paralysis of extremities. On laboratory examination, no hypoglycemia, electrolyte imbalance, or liver dysfunction was observed.

Cranial computed tomography demonstrated enlarged bilateral ventricles compared to those 3 years ago (Figure 1A,B), which also showed previously placed catheters in bilateral ventricles. Subsequently, she underwent chest and abdominal radiography; however, it could not disclose a catheter that should descend through the subcutaneous layers of the chest (Figure 2A). The abdominal radiograph demonstrated a reverse U‐shaped catheter that seemed to have been fractured in the left peritoneal cavity (Figure 2B). Thus, the diagnosis was ventriculoperitoneal shunt dysfunction due to catheter fracture, leading to aggravation of hydrocephalus. She underwent laparoscopic removal of the fractured catheter and a ventriculoperitoneal shunt revision for deteriorated hydrocephalus. The postoperative course was uneventful, and her mildly impaired consciousness improved promptly, while headache and nausea subsided.

**FIGURE 1:**
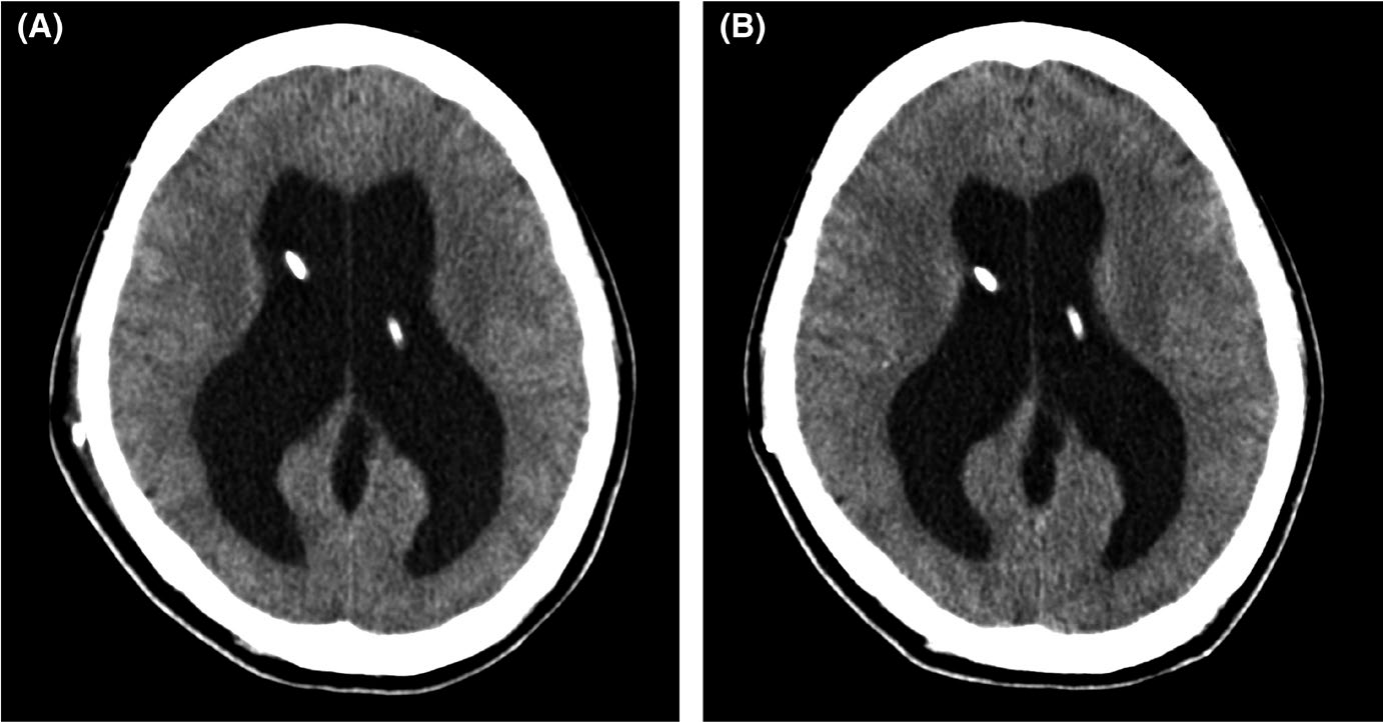
Cranial computed tomography demonstrating enlarged bilateral ventricles (A) compared to those 3 years ago (B) due to hydrocephalus and a ventriculoperitoneal catheter placed in the bilateral ventricles

**FIGURE 2:**
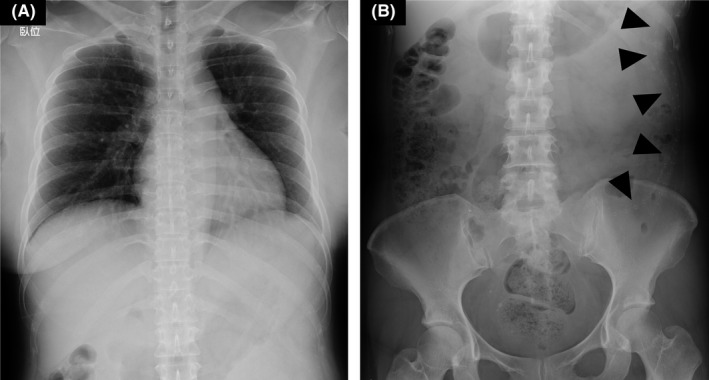
The chest radiograph showing no catheter that should descend subcutaneously through the chest (A), and he abdominal radiograph showing a fractured and migrated catheter in the left peritoneal cavity (B, black arrowheads)

The traction caused by growth during childhood can led to the fracture or disconnection of a catheter, which is one of the causes of mechanical ventriculoperitoneal shunt dysfunction as well as obstruction and infection of catheter. In this case, however, the last shunt revision was performed at the age of 20. Degeneration and calcification of the catheter can also cause shunt catheter fracture,^1^ and catheter fracture occurs most frequently in the neck.^2^ Therefore, cervical movement may have damaged the degenerated catheter.

Sometimes the shunt system continues to function despite radiologically suspected catheter fracture.^3^ However, radiographic fractures or disconnections of ventriculoperitoneal shunt catheter detected for the first time after the age of 15 years are always accompanied by shunt dysfunction, presenting with headache, vomiting, and impaired consciousness.^3^

This was considered to be an interesting case, as the cause of her symptoms could have been identified only through abdominal radiography findings, retrospectively.

## CONFLICT OF INTEREST

The authors declare no conflict of interests for this article.

## PATIENT CONSENT FOR PUBLICATION

Obtained.
